# Insights into Domain Organization and Regulatory Mechanism of Cystathionine Beta-Synthase from *Toxoplasma gondii*

**DOI:** 10.3390/ijms23158169

**Published:** 2022-07-25

**Authors:** Carolina Conter, Silvia Fruncillo, Filippo Favretto, Carmen Fernández-Rodríguez, Paola Dominici, Luis Alfonso Martínez-Cruz, Alessandra Astegno

**Affiliations:** 1 Laboratory of Biochemistry, Department of Biotechnology, University of Verona, Strada le Grazie 15, 37134 Verona, Italy; carolina.conter@univr.it (C.C.); silvia.fruncillo@postgrad.manchester.ac.uk (S.F.); filippo.favretto@univr.it (F.F.); paola.dominici@univr.it (P.D.); 2 Center for Cooperative Research in Biosciences (CIC bioGUNE), Basque Research and Technology Alliance (BRTA), Bizkaia Technology Park, Building 801A, 48160 Derio, Spain; cfernandez@cicbiogune.es (C.F.-R.); amartinez@cicbiogune.es (L.A.M.-C.)

**Keywords:** cystathionine beta-synthase, *Toxoplasma gondii*, Bateman module, mutagenesis, enzyme activation, oligomerization, folding

## Abstract

Cystathionine beta-synthase (CBS) is a key regulator of homocysteine metabolism. Although eukaryotic CBS have a similar domain architecture with a catalytic core and a C-terminal Bateman module, their regulation varies widely across phyla. In human CBS (HsCBS), the C-terminus has an autoinhibitory effect by acting as a cap that avoids the entry of substrates into the catalytic site. The binding of the allosteric modulator AdoMet to this region alleviates this cap, allowing the protein to progress from a basal toward an activated state. The same activation is obtained by artificial removal or heat-denaturation of the Bateman module. Recently, we reported the crystal structure of CBS from *Toxoplasma gondii* (TgCBS) showing that the enzyme assembles into basket-like dimers similar to the basal conformers of HsCBS. These findings would suggest a similar lid function for the Bateman module which, as in HsCBS, should relax in the absence of the C-terminal module. However, herein we demonstrate that, in contrast with HsCBS, removal of the Bateman module in TgCBS through deletion mutagenesis, limited proteolysis, or thermal denaturation has no effects on its activity, oligomerization, and thermal stability. This opposite behavior we have now found in TgCBS provides evidence of a novel type of CBS regulation.

## 1. Introduction

Cystathionine β-synthase (CBS) is a pyridoxal phosphate (PLP)-dependent enzyme that catalyzes the β-replacement of serine (L-Ser) and homocysteine (L-Hcys) to produce cystathionine (L-Cth) in the reverse transsulfuration pathway. L-Cth is then converted to cysteine (L-Cys) by a second PLP-dependent enzyme, the cystathionine γ-lyase (CGL). Alternatively, CBS can use the PLP cofactor to catalyze the condensation of L-Cys with L-Hcys forming L-Cth and hydrogen sulfide (H_2_S), a gaseous signaling molecule involved in mediating significant physiological effects [1].

**Figure 1:**
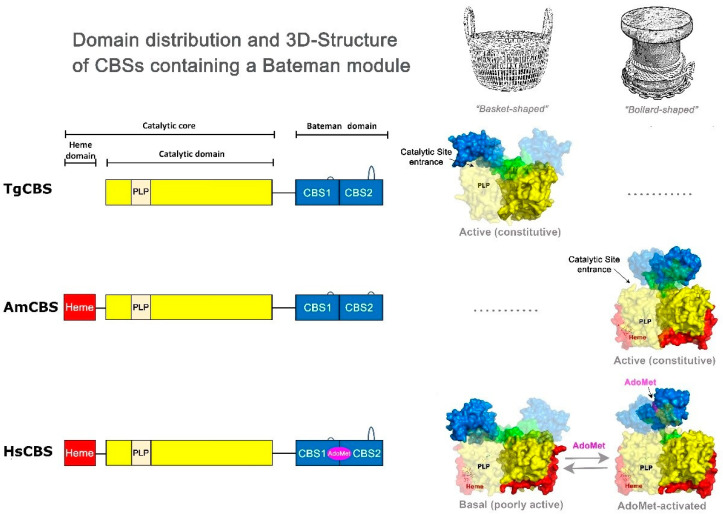
Three-dimensional structure of CBS enzymes containing a Bateman module. Domain distribution in CBSs containing a Bateman module (left). The more complex architecture is found in HsCBS (**bottom**) and contains a N-terminal heme-binding domain (red), a catalytic domain that hosts the PLP cofactor (yellow), and a C-terminal Bateman module (blue) that can host AdoMet (magenta). The interdomain linker is in green. The two complementary subunits of each dimer are represented by opaque and transparent surfaces, respectively. DmCBS or AmCBS contains the same domain architecture found in HsCBS but is not regulated by AdoMet (**middle**). TgCBS lacks the heme-binding domain and is not allosterically regulated by AdoMet (**top**).

CBS adopts a multidomain architecture that differs across organisms and is best exemplified in the well-studied human enzyme [2,3,4,5]. Human CBS (HsCBS) contains: the N-terminal heme-binding region, which was suggested to function in enzyme folding and/or redox sensing [6,7,8,9]; a central catalytic core which is highly conserved in the fold type II PLP enzymes [2,10]; and the C-terminal *S*-adenosyl-L-methionine (AdoMet) binding regulatory domain [11,12,13,14]. The binding of the allosteric modulator AdoMet causes a shift in the enzyme conformation from a basket-shaped low activity basal state [15] to a marine mooring bollard-shaped more active conformation [5,16]. In contrast, only a single conformation which is constitutively activated has been found in less evolved organisms, such as *Drosophila melanogaster* (DmCBS) and *Apis mellifera* (AmCBS). Structurally, this conformation is equivalent to the HsCBS in the AdoMet-bound form. CBS from *Saccharomyces cerevisiae* (ScCBS) shows a less elaborated organization, lacking the N-terminal domain and being unresponsive to AdoMet [4,17]. Most bacteria possess the simplest domain architecture, lacking both the heme and the C-terminal Bateman domain [18,19,20] (Figure 1).

Based on these differences in domain architecture, it is crucial to attribute specific functions to the various regions of CBS and to know how the interactions between them modulate the activity of the enzyme. One way to shed light on the structure and regulation of this unique enzyme is to adopt an evolutionary approach, searching for non-canonical CBS enzymes from different organisms. *Toxoplasma gondii*, the causative agent of toxoplasmosis, possesses a functional CBS (TgCBS). Previous structure-function analyses of the recombinant TgCBS have provided significant insights into the kinetic properties and domain organization of this protein. It was determined that TgCBS can convert both L-Ser and O-acetylserine (L-OAS) to L-Cth as well as condensate L-Cys with L-Hcys to efficiently generate H_2_S [21]. In contrast to HsCBS, TgCBS does not possess an N-terminal heme binding region and is unresponsive to AdoMet, despite containing a C-terminal Bateman module (Figure 1). Notably, the three-dimensional structure of a TgCBS construct missing a few residues from an internal loop (which are not involved in the activity or the oligomerization state, TgCBS Δ466–491) revealed that the enzyme exists in an active basal-type (basket-shaped) folding, which likely constitutes its sole conformational state [22]. These findings raise critical new questions about whether CBSs with similar assemblies are always (or not) subject to evolve allosterically towards a second activated conformation. Aimed to shed light on this matter, herein we have used deletion mutagenesis of the C-terminal Bateman domain, limited proteolysis, and thermal denaturation as multiple approaches to expand our knowledge of the mechanism of TgCBS regulation. It is well-known that the C-terminal domain of HsCBS has a profound influence on the oligomerization, stability, and activity of the enzyme [3,4]. The activity of HsCBS can be stimulated in vitro by different processes: by binding of the allosteric modulator AdoMet [23], by removal of the C-terminal domain via treatment with trypsin or protein engineering [24], or by heat activation [4,23]. All these processes determine a similar level of HsCBS activation, indicating that a common mechanism comprising the displacement of the Bateman domain from its site of inhibition underlies these different forms of activation. We found that the elimination of the C-terminal Bateman module in TgCBS through deletion mutagenesis, limited proteolysis, and thermal denaturation does not affect either the oligomerization or the stability of the enzyme and, most importantly, has no effects on TgCBS activity, indicating that the commonly accepted role of the Bateman domain as a lid that gives, or alternatively impairs, the access of substrates into the catalytic cavity, is not met by TgCBS. Overall, our data support the existence of a new type of CBS folding, structurally related to the basal form of HsCBS, with different activity and regulation.

## 2. Results

### 2.1. Production of Recombinant TgCBS Lacking the C-Terminal Domain

**Figure 2:**
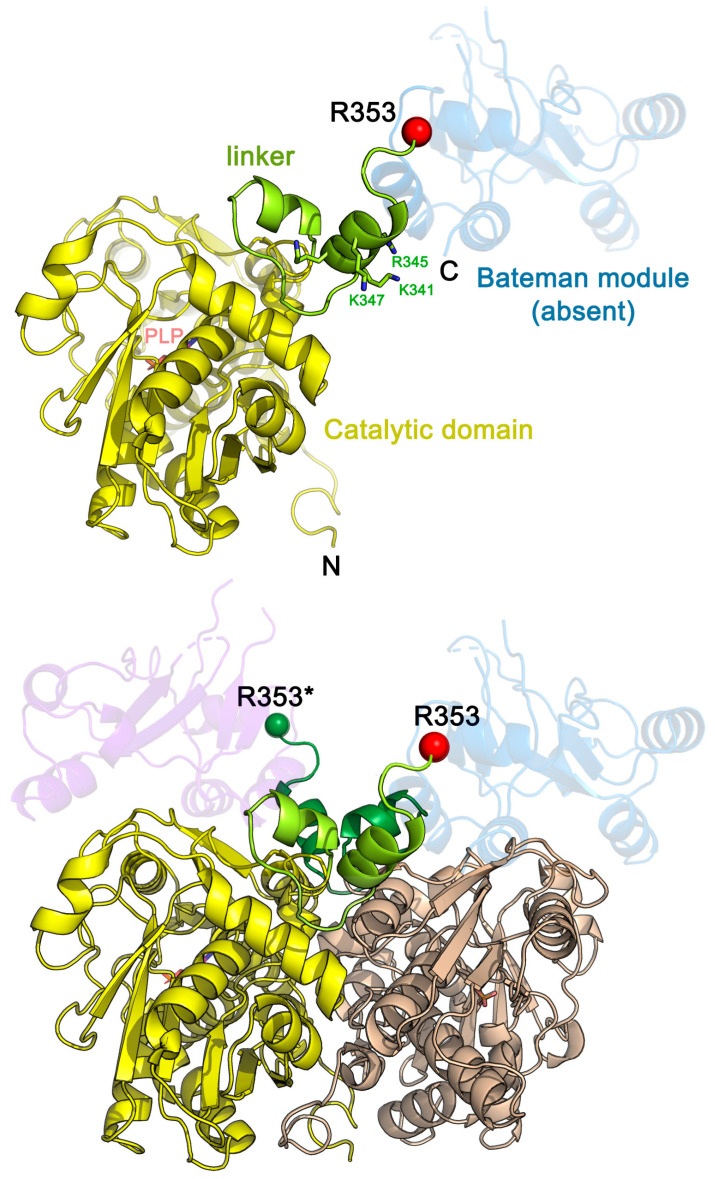
Structure of TgCBS R353*. Three-dimensional structure of the monomeric (**up**) and dimeric (**down**) construct R353* extrapolated from the crystal structure of TgCBS [22]. PLP and potential target residues of trypsin-cleavage are in sticks. The location of residue R353 in each monomer is indicated in red and green spheres. In the dimer, the catalytic domains of the complementary subunits are colored yellow and light brown, respectively. The Bateman modules (absent in the R353* construct) are represented in transparent ribbons (colored in blue and wine).

As a first approach to studying the mechanism of TgCBS regulation, we produced a recombinant enzyme lacking the C-terminal region (residues 353–514) by introducing a STOP codon at position R353 in the sequence of the enzyme (TgCBS R353*). Residue 353 is located at the end of the interdomain linker, connecting the catalytic domain with the Bateman module. More concretely, R353 is placed just before the first short helix of the Bateman module (Figure 2). Accordingly, the construct R353* structure is not expected to be limited and/or structurally affected by the main chain designed truncation. Of note, previous constructs engineered on the HsCBS [2,6] and ScCBS [17], in which the polypeptide chain had been truncated at more compromised positions, closer to the end of the catalytic domain, were shown not to interfere with either the structure or the activity of the resulting proteins. The truncated R353* construct yielded a soluble enzyme which was successfully purified to homogeneity (>95%, Figure S1), with a 6xHis tag at the N-terminus. The C-terminally truncated 39 kDa enzyme showed a UV-Vis absorption spectrum with a dominant peak at 411 nm, which represents the ketoenamine form of the internal aldimine, as observed for the full-length enzyme (Figure S1). A stoichiometry of 0.9 ± 0.1 PLP per monomer of TgCBS R353* was found. Thus, no differences were observed in the UV-Vis absorption spectrum compared to the full-length enzyme.

**Figure 3:**
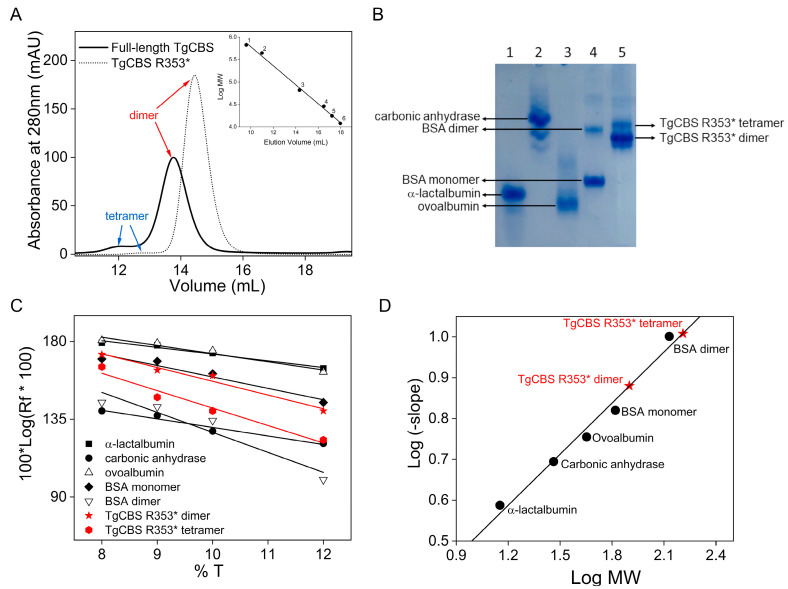
Oligomeric state of TgCBS R353*. (**A**) Gel filtration chromatography of full-length TgCBS (solid line) and TgCBS R353* (dotted line) at 1 mg/mL using a Superdex 200 10/300 GL column in 20 mM sodium phosphate buffer pH 8.5, 150 mM NaCl and 0.1 mM DTT. Inset, calibration curve of the logarithm of the molecular weight versus elution volumes. The standard proteins used were: 1, thyroglobulin; 2, apoferritin; 3, albumin bovine serum (BSA); 4, carbonic anhydrase; 5, myoglobin; 6, cytochrome c. (**B**–**D**) Relative electrophoretic mobility calculation of TgCBS R353* dimer and tetramer by Ferguson plot. (**B**) Representative native-PAGE (8% T) showing the mobility of standard proteins and TgCBS R353*. Lane 1: α-lactalbumin; Lane 2: carbonic anhydrase; Lane 3: ovoalbumin; Lane 4: BSA (dimer and monomer); Lane 5: TgCBS R353*. (**C**) Effect of %T on the relative mobility of standard proteins and TgCBS R353*. Plot of 100 [log (Rf × 100)] versus %T, according to Ferguson et al. [27]. (**D**) Standard curve was obtained by plotting the log of the negative slope (from (**C**)) against the log of standard proteins’ molecular weight.

Size exclusion chromatography (SEC) and native PAGE analysis were used to monitor the effect of truncation on the oligomeric status of TgCBS (Figure 3). It is known that the elimination of the C-terminal domain goes along with a change in oligomeric status from a mainly tetrameric to a mainly dimeric form in the case of HsCBS and ScCBS [25,26], while DmCBS forms native dimers both in the presence and absence of this domain [3]. In the case of TgCBS, the removal of the C-terminal domain does not lead to major changes in the oligomeric status. Indeed, the C-terminally truncated enzyme was eluted in SEC as one larger peak corresponding to a dimeric species and a minor peak to a tetrameric form (dimer and tetramer populations were 97% and 3%, respectively). The same behavior was previously observed for full-length TgCBS (92% dimer and 8% tetramer) (Figure 3A) [21,22]. These results were further confirmed by native PAGE analysis of TgCBS R353* at various polyacrylamide concentrations (8, 9, 10, and 12%). Figure 3B shows the 8% gel as representative of the observed electrophoretic profiles. By Coomassie staining, a pattern characterized by the presence of two bands was revealed, with the faster-migrating species being dominant. Ferguson plot analysis [27] was used to analyze data from native gels (Figure 3C,D). Accordingly, the molecular mass of the two bands was shown to be 79 and 141 kDa. Thus, the major band could be concluded to correspond to protein dimers (79/39 kDa, ~2), whereas the minor band originated from protein tetramers (141/39 kDa, ~4). This data confirmed that the removal of the C-terminal domain does not affect the oligomeric state of TgCBS, and the dimeric species is the predominant species in solution for both the full-length and the C-terminally truncated enzyme.

**Figure 4:**
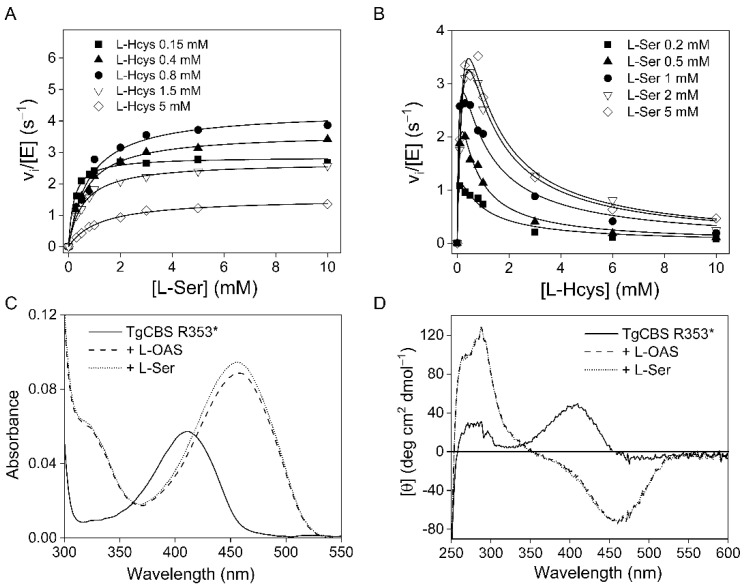
Canonical activity of TgCBS R353*. (**A**,**B**) Steady-state initial velocity kinetics for TgCBS R353* showing the dependence of the reaction on [L-Ser] and on [L-Hcys]. (**A**) The concentration of L-Ser was varied at a fixed concentration of L-Hcys. (**B**) The concentration of L-Hcys was varied at a fixed concentration of L-Ser. The fit of the entire data set to Equation (1) is represented by the lines. (C-D) Spectra of TgCBS R353* in the presence of substrates. (**C**) Absorbance spectra of 10 µM TgCBS R353* alone (solid line) and in the presence of 10 mM L-OAS (dashed line) or 10 mM L-Ser (dotted line). (**D**) CD spectra of 1 mg/mL TgCBS R353* alone (solid line) and in the presence of 10 mM L-OAS (dashed line) or 10 mM L-Ser (dotted line).

The canonical activity of TgCBS following removal of its C-terminal extension was then compared to that of the full-length protein by applying the previously described CBL-LDH assay [21,28,29]. Figure 4A,B shows the sample data for the condensation of L-Ser and L-Hcys to L-Cth performed by TgCBS R353*. As previously reported, substrate inhibition by L-Hcys is present. We found no significant differences in the ability of full-length TgCBS and TgCBS R353* to catalyze the β-replacement reaction (Table 1), revealing that the removal of the Bateman module does not impact on the enzyme activity of the TgCBS. In contrast, truncation of the same domain results in ~5-fold and ~2-fold activation for the HsCBS [30] and ScCBS [26], respectively. The same experiments were performed using L-OAS as a substrate and, again, no major differences in kinetic parameters between the full-length and truncated enzyme were found (Table 1). Moreover, the addition of L-Ser or L-OAS resulted in the disappearance of the internal aldimine band (411 nm) and the concomitant appearance of the aminoacrylate band (460 nm) in both absorbance and CD spectra (Figure 4C,D), as already observed for the full-length TgCBS [21,22].

### 2.2. Trypsin Cleavage of Recombinant TgCBS

**Figure 5:**
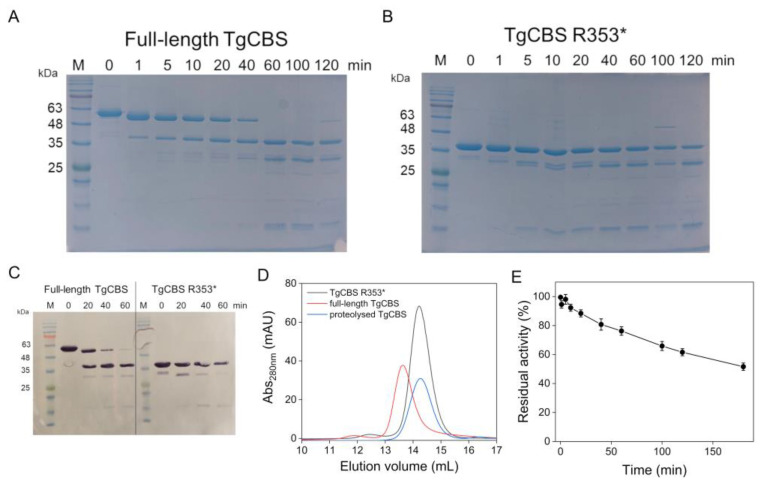
Trypsin cleavage of TgCBS. (**A**,**B**) Representative time course of trypsin limited proteolysis of (**A**) full-length TgCBS and (**B**) TgCBS R353*. Lane 1: MW marker, lanes 2–9: trypsin digestion products obtained following incubation of protein with trypsin 1:200 (*w/w*) for 0, 1, 5, 10, 20, 40, 60, 100, and 120 min, respectively. (**C**) Western blot analysis after trypsin treatment. At various time, 0, 20, 40, 60 min, proteolyzed products were immunoblotted with anti-His antibodies (Sigma, 1:2000). (**D**) SEC of TgCBS after limited digestion (blue line). Full-length TgCBS (red line) and TgCBS R353* (black line) at 0.5 mg/mL are also shown. (**E**) Time-course of TgCBS activity during trypsinolysis.

Limited proteolysis of TgCBS was performed as an alternative approach to study its regulation mechanism. Limited trypsin digestion of native TgCBS monitored by SDS-PAGE showed the gradual cleavage of the original ~57 kDa subunit (representing the monomer of the intact enzyme) into two main ~56 kDa and ~39 kDa proteolytic products (Figure 5A). The 56 kDa product decreased rapidly within the first 60 min of the reaction, while the 39 kDa species was extremely stable and resistant to further digestion since it did not disappear even at longer incubation times. This species reacted with an antibody raised against the N-terminal His-tag (Figure 5C), indicating that it contains the extreme N-terminal region of TgCBS. Parallel experiments on the TgCBS R353* variant suggested that the recombinant truncated enzyme behaves like the above described proteolytically resistant core of full-length TgCBS. Indeed, a pattern characterized by the presence of one main 39 kDa band, corresponding to the TgCBS R353* enzyme monomer, which is resistant to digestion and continues to accumulate up to 120 min, was observed (Figure 5B). These results suggest that the PLP-containing catalytic domain represents the proteolytically resistant core and that limited proteolysis of full-length TgCBS results in the elimination of the C-terminal domain. To support these conclusions, we determined the native sizes of the proteolytic cleavage products by stopping the trypsinolysis after 120 min with a two-fold weight excess of soybean trypsin inhibitor and separating the mixture by SEC. The proteolysed full-length TgCBS eluted at the same volume as the C-terminally truncated TgCBS R353* as a species of approximately 78 kDa (a dimer of 39 kDa subunit) (Figure 5D), thus implying that proteolyzed TgCBS lacks the C-terminal domain and TgCBS has a stable, protease-resistant core of 39 kDa.

To investigate the effect of the removal of the C-terminal domain by trypsin on TgCBS activity, we followed the time course of TgCBS activity during trypsin digestion. The proteolytic cleavage of TgCBS was not accompanied by an increase in enzyme activity (up to 180 min) (Figure 5E). Thus, while in HsCBS removing the C-terminal regulatory domain by limited proteolysis, results in a more active enzyme and the conversion of a tetramer to a dimer of 45 kDa subunits [24], no significant effects were observed in TgCBS.

### 2.3. Thermal Denaturation

**Figure 6:**
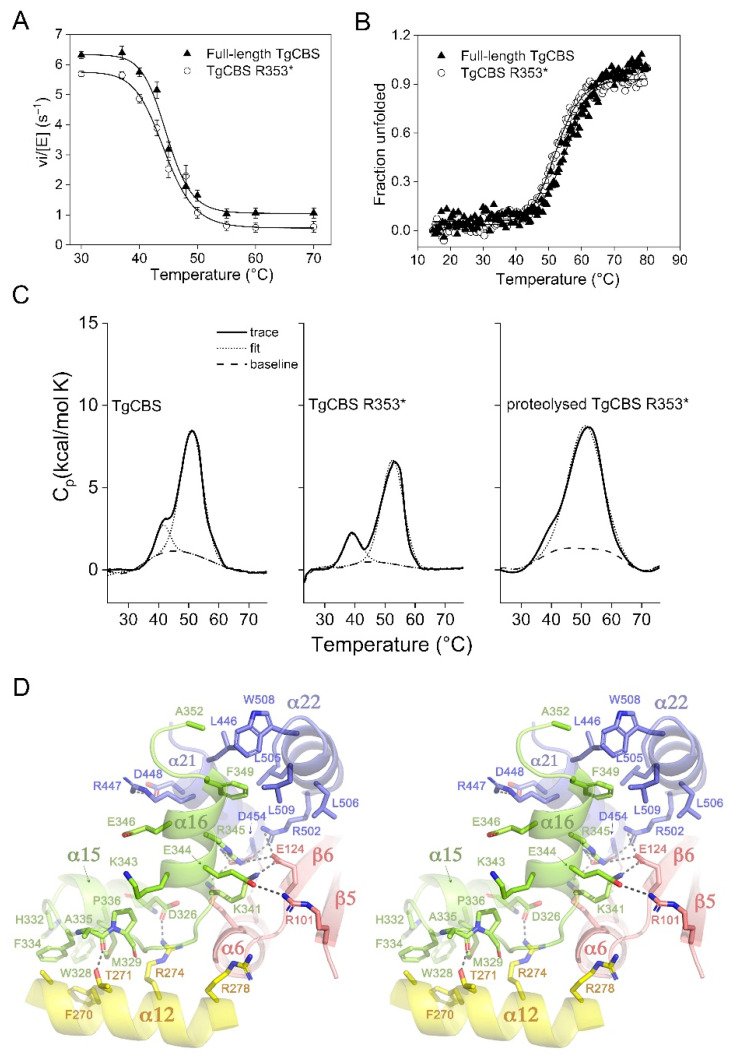
Thermal denaturation of TgCBS variants. (**A**) Effect of thermal pre-treatment of full-length TgCBS (solid triangles) and TgCBS R353* (open circles) on their steady-state initial velocity at saturating concentration of L-Ser and L-Hcys in the canonical reaction. Each data set is relative to four replicas and error bars represent S.E.M. (**B**) Thermal denaturation of 0.2 mg/mL full-length TgCBS (solid triangles) and TgCBS R353* (open circles) recorded following ellipticity signal at 222 nm in 20 mM sodium phosphate buffer pH 8.5. (**C**) Thermal denaturation of proteins analyzed by DSC. The solid lines show the row traces, the dashed lines show the chemical baseline whereas the dotted lines show fittings for each transition to a two-state irreversible denaturation model. The scan rate was 0.8 °C/min and the protein concentration was 30 μM in the protein subunit. (**D**) Stereo pair showing the main interactions existing between the interdomain linker and the protein domains in TgCBS. Residues belonging to the TgCBS linker, the catalytic domain and the Bateman module are in green, yellow, and blue, respectively. Residues and elements colored in light brown belong to the catalytic domain of the complementary subunit.

It has been demonstrated that a progressive thermal denaturation of HsCBS causes the activation of the enzyme (by approximately three-fold) because of the irreversible denaturation of the C-terminal regulatory domain [31]. The denaturation of the regulatory Bateman module releases the occlusion exerted by this module on the entrance of the active site, and thus mimics the stimulation by AdoMet, which, upon binding, induces a conformational change that triggers the same relief effect. Accordingly, an HsCBS mutant lacking the C-terminal regulatory region (Δ414−551), which is constitutively activated, is significantly resistant to heat denaturation and its catalytic activity is not altered up to 60 °C [4]. To explore whether TgCBS activity is affected in vitro by heat denaturation, the canonical L-Ser CBS-dependent activity of TgCBS R353* was examined after 10 min at different temperatures (30–70 °C) and compared with that of full-length TgCBS (Figure 6A). We found no significant changes in the enzyme activity profile of TgCBS R353* upon heat treatment (T_50_ of 44.3 ± 0.6 °C) compared to the full-length TgCBS (T_50_ of 44.6 ± 0.3 °C), supporting the findings above, that removal of the C-terminal module does not modify the enzyme activity.

We next probed the thermal stability of TgCBS variants following the protein secondary structure by heating the sample up to 90 °C via CD spectroscopy. As shown by the temperature dependence of the CD signal at 222 nm (Figure 6B), TgCBS R353* unfolds cooperatively during thermal denaturation, with a single mid-point temperature of 52.5 ± 0.2 °C. A cooperative transition was also observed for the full-length TgCBS even with a slightly higher mid-point temperature (56.3 ± 0.5 °C). This difference could be due to the absence in TgCBS R353*of the CBS1 and CBS2 domains, which have the βαββα secondary structure elements usually present in these motifs [12]. To study the thermal stability of TgCBS in more detail, we employed DSC (Figure 6C). The DSC traces were scan rate dependent and irreversible (data not shown). Denaturation of the full-length TgCBS resulted in the presence of one main peak with a T_m_ of 51.1 ± 0.2 °C and ΔH of 80 ± 9 kcal/mol and a shoulder at 42.3 ± 0.7 °C with ΔH of 16 ± 5 kcal/mol (Figure 6C and Table S1). The unfolding of TgCBS R353* was characterized by two separate peaks on the thermogram with T_m_ values of 39.2 ± 0.1 and 52.7 ± 0.1 °C and a molar enthalpy of Δ*H* = 21 ± 7 and 67 ± 2 kcal/mol, respectively (Figure 6C and Table S1). Theoretical ΔH values representing denaturation of only the catalytic domain (residues 1–323), the catalytic domain including the linker (residues 1–352, corresponding to the TgCBS R353*), and the catalytic domain, linker and C-terminal domain together (residues 1-514, corresponding to the intact TgCBS) are 225, 246, and 359 kcal/mol, respectively [4,32]. Given that the absence of the C-terminal domain in the truncated enzyme has no effect on the main-temperature transition and that TgCBS variants are almost completely inactive at denaturation transitions by DSC (~52 °C) (Figure 6A), we assume that the main thermal transition in the two proteins represents the denaturation of at least the catalytic domain. However, based on the comparison between the experimental and theoretical unfolding enthalpy in the case of full-length TgCBS, we cannot say if only the catalytic core was unfolding or if both catalytic and C-terminal domains partially unfolded. The DSC profile of the trypsin cleaved TgCBS R353* did not show a distinct peak at 39 °C, although no significant changes in the T_m_ value of the main transition were noted (T_m_ of 51.2 ± 0.1 °C) (Figure 6C), suggesting that the low 39 °C temperature transition could represent the unfolding of an exposed region susceptible to proteolysis. The proteolytic product has an intact extreme N-terminus given that it was detected by the antibody specific for the N-terminus His-tag (see above). Thus, the 39 °C peak may represent the denaturation of the linker (residues 324–352) connecting the two functional domains (catalytic and C-terminal) and is likely more masked in the calorimetric traces of full-length TgCBS (Figure 6C). The ΔH of the peak at 39 °C closely matches the theoretical ΔH value for linker region unfolding (19.5 kcal/mol). A close view of the crystal structure of TgCBS [22] shows that the interdomain linker folds on itself and is fixed by a combined set of interactions at its two extremes, with both the catalytic domain and the Bateman module (Figure 6D). Beyond reducing the mobility of the linker, these contacts help to configure a sole protein structural body with almost no mobile elements, which is consistent with a single peak in the thermal denaturation event in the full-length TgCBS protein. Interestingly, and despite sharing a similar overall basket-like fold in the absence of AdoMet, the HsCBS [22] shows a less restricted linker, an essential feature to allow the displacement of the Bateman module when binding the allosteric activator.

### 2.4. Structural Analysis of Cavities in TgCBS

**Figure 7:**
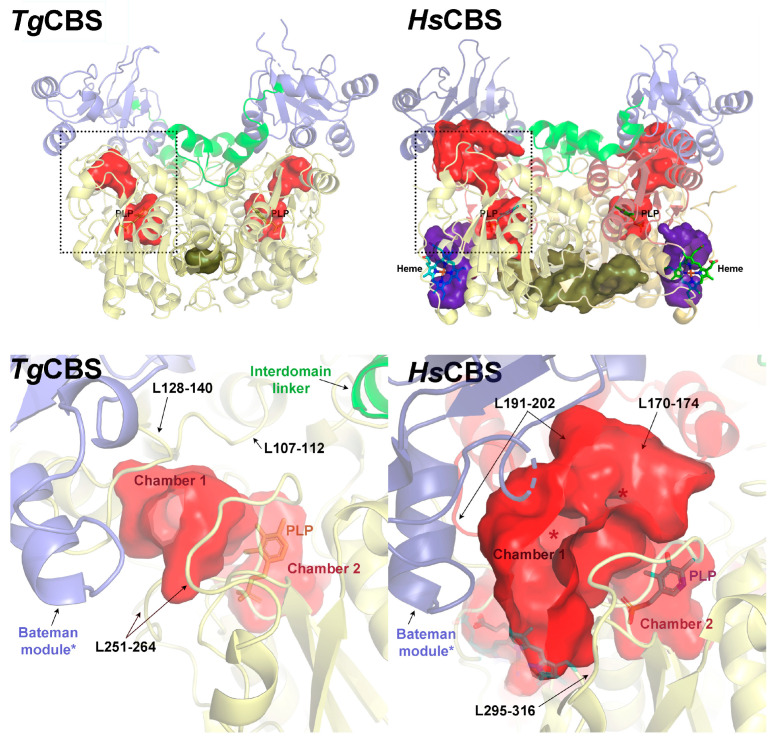
Catalytic cavity of TgCBS and HsCBS. The picture shows the main structural elements defining the entrance of the catalytic cavity in TgCBS (**left**) and HsCBS (**right**). The location of the main cavities within the corresponding CBS basket-like protein dimers are framed in dotted lines and zoomed up in the lower panels. The complementary catalytic cores, interdomain linkers and Bateman modules are represented by *yellow*, *green,* and *dark blue* ribbons, respectively. The interior surfaces are colored (*red* for the catalytic cavity, *green* for the intersubunit cavity, and *violet* for the heme-binding cavity (the latter is absent in TgCBS as the native protein lacks a heme-binding domain). The different entries into the first chamber of the catalytic cavity, which is exposed to the solvent, are indicated with asterisks. Chamber-2, located underneath and connected to Chamber-1, is deeply buried in the protein core and hosts the PLP cofactor (in sticks).

To shed some light on the constitutively active, basket-like fold that we have identified in TgCBS, we analyzed the size, shape, and features of the internal cavities present in the enzyme and compared it with its CBS homologs (Figure 7 and Figure S2). We found that, beyond the large cleft configuring the heme-binding domain in mammals and insects’ CBS (colored in violet in Figure S2), obviously not present in TgCBS (Figure 7), the main cavity hosting the PLP cofactor is narrower and less bulky in TgCBS (Figure 7 and Figure S2). Interestingly, the catalytic cavity of TgCBS is isolated and disconnected from the rest of the clefts present in the protein (catalytic cavity in red, intersubunit surface cleft in green, and heme-binding cleft surface in violet in Figure S2), being accessible from the exterior by a sole entrance delimited by the CBS1 motif and three loops belonging to the catalytic core, including amino acid residues 107–112, 128–140, and 251–264. Another intriguing feature is that the catalytic site is divided into two well-differentiated chambers that are connected to each other (Figure 7). Chamber-1 is the only one exposed to the solvent, while chamber-2 is deeply buried in the protein and hosts the PLP cofactor. Like TgCBS, the human enzyme features two well-distinguished chambers. However, HsCBS shows a wider entrance to the first compartment, which shows at least two potential entry points (highlighted with asterisks in Figure 7) that may determine the access of the different substrates. As in TgCBS, the second cubicle is buried and protected from the solvent. The precise delineation of the catalytic cavity that characterizes the basket-shaped dimeric species seems to be blurred in the bollard-type CBS dimeric assemblies, which, in addition to widening at the entrance, are interconnected at the top through a wide tunnel running between the CBS module (two antiparallel ensembled Bateman modules) and the catalytic core (Figure S2). The formation of such a tunnel is exclusive of the bollard-like CBS dimers (c.a AmCBS and DmCBS) and is possible thanks to the presence of the CBS module above the catalytic cores. Although its function is not yet known, we postulate that this tunnel could potentially provide a different pathway and represent a selective filter for different substrates (or an alternative exit pathway for the corresponding reaction products).

## 3. Discussion

Even though eukaryotic CBSs have a similar domain architecture with a catalytic core and a tandem of CBS motifs in the C-terminal region, the removal of their C-terminal domain has significantly different effects on the proteins, supporting the notion that the molecular mechanisms regulating the activity of CBS and the function exerted by the Bateman module vary widely across phyla. In humans, the enzyme activity is modulated by the crosstalk between the C-terminal regulatory domain and the catalytic core. Indeed, the C-terminus has an autoinhibitory effect by acting as a cap that avoids the entry of substrates into the catalytic site. This capping effect is alleviated by the binding of AdoMet to the Bateman module by inducing a relative rotation of its two CBS domains, which in turn weakens their interaction with the catalytic core, leaving the PLP cofactor exposed and allowing the protein to progress from a basal toward an activated state. At present, the exact pathway remains unknown followed by the Bateman module to subsequently dock with the same part of the complementary subunit to fix the active conformation in the mammalian enzyme. Removal of the regulatory module or alternatively, a thermal treatment, alleviates the occlusion exerted by the Bateman module and activates HsCBS, permitting unrestricted access of substrates into the catalytic center [4,23,24]. In contrast, the crystal structure of DmCBS, which is constitutively locked in an activated state similar to the AdoMet-bound activated conformation of HsCBS, does not apparently exhibit any significant communication of the catalytic core with the regulatory domain [3,33]. Interestingly, truncation of DmCBS yielded insoluble protein, indicating that the Bateman module is necessary to achieve a soluble fold.

Recently, we found that TgCBS forms constitutively active dimers, unresponsive to AdoMet, where the two complementary Bateman modules are distant from each other, as in the basal state of HsCBS, and are arranged over the catalytic cavity. These findings would suggest a similar lid function for the Bateman module which restricts the free entry of substrates into the catalytic cleft and which, as proved in HsCBS, should relax in the absence of the C-terminal module. Surprisingly, here we found that the commonly accepted role of the Bateman domain as a lid that gives, or alternatively impairs, the access of substrates into the catalytic cavity is not met by TgCBS. Indeed, the removal of the Bateman module of TgCBS through deletion mutagenesis, limited proteolysis, and thermal denaturation has no effect on protein activity. This opposite behavior that we have now found in TgCBS not only unravels major differences between the apicomplexan and mammalian CBS enzymes but highlights the role/s of the Bateman domain in different CBSs remains poorly understood and requires further research efforts. Moreover, these findings help to answer one major question raised in one of our previous studies, whether TgCBS might alternatively be activated by other adenosyl derivatives, or molecules, different from AdoMet. In such a hypothetical case, even if the allosteric effector remains unidentified, the artificial removal of the Bateman domain should result in a significantly more active species, which, as shown now, is not observed. Thus, our new data supports the existence of a sole basket-like conformation, not capable of progressing towards a second conformer where the complementary Bateman modules would interact with each other.

Supporting the existence of a novel type of CBS fold, DSC experiments reveal thermal denaturation profiles for TgCBS variants that are markedly different from those reported for HsCBS and DmCBS (Table S1). Thermal denaturation curves of HsCBS exhibited two endothermic peaks with T_m_ values of ~53 °C and ~70 °C that are attributed to the C-terminal regulatory domain and the catalytic domain, respectively [4,31] (Table S1). Accordingly, the DSC profiles of HsCBS in the presence of AdoMet and the truncated mutant HsCBSΔ414–551 (which lacks the Bateman domain) are characterized by the absence of the low-temperature transition [31] (Table S1). The unfolding of DmCBS displays only one peak with a T_m_ of ~70 °C, revealing that its regulatory domain is largely stabilized similar to the stabilization effect of AdoMet on HsCBS (Table S1). These results support the notion that AdoMet-bound HsCBS and DmCBS exist in a conformation where the regulatory CBS domains form a compact disk-like CBS module clearly separated from the catalytic core. Interestingly, the single transition observed in both full-length TgCBS and TgCBS R353* with a T_m_ of ~52 °C suggests the presence of a sole structural body with almost no mobile elements and supports a basket-like arrangement of TgCBS. The sole structural conformation is also supported by the reduced mobility of the linker observed in the 3D structure which folds on itself and is fixed by a combined set of interactions at its two extremes with both, the catalytic domain, and the Bateman module. Once the Bateman module is removed, the linker barely interacts with the core in the mutant R353*, whereas the linker is tightly attached to the Bateman in the full-length protein. This could explain why the linker may give a separate unfolding peak in the R353* truncated construct. Based on our DSC results, it is possible that the T_m_ value represents a good biophysical marker to analyze in solution the regulation mechanism of CBS enzymes. Moreover, our new findings might pave the way to predicting the orientation of the Bateman domain in other CBSs.

Importantly, discovering that TgCBS exists as a single constitutively active, basket-like fold [21,22], invites us to rethink the structural evolution of CBS enzymes. Our findings underline that for some CBS enzymes containing a C-terminal Bateman module, the conformational transition from a basket- to a bollard-like folding is neither an essential nor a necessary condition to become functionally active. This new scenario suggests that the basket-like conformation is evolutionarily older than the bollard-like dimeric assembly. Moreover, the basal basket-fold found in HsCBS (poorly active) might have evolved from an active basket-like arrangement like the one found in TgCBS. Based on our new data, we propose that the basket-like arrangements of CBSs should, in turn, be subdivided into two subclasses: (i) constitutively active species and (ii) allosterically regulated assemblies.

Overall, the data we now present in this study and in our previous works [21,22] highlight the relevance of a comprehensive multidisciplinary characterization of different CBSs, in order to accurately determine their conformational universe throughout evolution. Moreover, they should contribute significantly to the design of effective modulators of this enzyme in different organisms in the coming years.

## 4. Materials and Methods

### 4.1. Protein Production

Full-length TgCBS was expressed in *E. coli* Rosetta (DE3) upon induction with 0.5 mM isopropyl-β-D-1-thiogalactopyranoside at 24 °C for 15 h and purified with a tag of six His at the N-terminal using a Ni-sepharose column as previously described [21]. TgCBS lacking the C-terminal domain (TgCBS R353*) was produced by site specific mutagenesis on the pET21a-TgCBS construct, using the QuikChange^®^ site-directed mutagenesis kit (Agilent Technologies), according to the manufacturer’s recommendations, introducing a STOP codon in position R353. The presence of the desired mutation was confirmed by DNA sequencing. The verified plasmid for truncated TgCBS was transformed into *E. coli* Rosetta (DE3) expression host cells and the corresponding protein was purified as the wild-type enzyme [21]. The purity of the enzyme was confirmed by SDS-PAGE. Proteins were flash frozen in liquid N_2_ and stored at −80 °C until use. The PLP content was determined by treating the enzyme with 0.1 M NaOH and measuring absorbance at 388 nm [34].

### 4.2. Steady-State Kinetics

The CBS-catalyzed condensation of L-Ser (or L-OAS) and L-Hcys to produce L-Cth was assayed via the continuous CBL-LDH assay, as previously described [21,28,35]. In brief, enzyme activity was measured using a Jasco V-560 UV-visible spectrophotometer in a total volume of 200 µL at 37 °C. Reactions were carried out in assay buffer (50 mM MOPS, 50 mM bicine, 50 mM proline (MBP) pH 9) containing 0.2 mM NADH, 1.5 μM CBL, 2 μM LDH, 0.1–30 mM L-Ser (or 1–100 mM L-OAS), and 0.05–10 mM L-Hcys. These were initiated by the addition of 0.2–2 μM TgCBS. A background rate, for all components except the TgCBS enzyme, was recorded for each sample before initiating the reaction by the addition of the enzyme.

For gradual thermal denaturation, TgCBS variants were incubated at temperatures between 30–70 °C for 10 min, cooled on ice for 5 min, and then residual enzymatic activity toward L-Ser was assayed using the continuous CBL-LDH assay.

Kinetic parameters were calculated via global analysis as previously described [21,26,35] from the fit of the data to Equation (1):

(1) $$\frac{v}{E}=\frac{k_{cat}*\left[SA\right]*\left[SB\right]}{K_{m}^{SB}*\left[SA\right]+K_{m}^{SA}*\left[SB\right]*\left(1+\frac{\left[SB\right]}{K_{i}^{SB}}\right)+\left[SA\right]*\left[SB\right]}$$

where *v* is the initial velocity, *E* is the concentration of the enzyme, *SA* is the concentration of the first substrate, *SB* the concentration of the second substrate, and *k_cat_* and *K_m_* are the catalytic and Michaelis–Menten constants, respectively. *K_i_^SB^* represents the inhibition constant for substrate inhibition by L-Hcys. Kinetic data are presented as the mean ± standard error of the mean (SEM) of three to five repetitions using at least three independently purified protein batches. Data fitting was carried out with OriginPro8 (OriginLab) software.

### 4.3. Spectroscopic Measurements

Absorption spectra were recorded on a Jasco-V560 UV-visible spectrophotometer using 1 cm path length quartz cuvettes in 20 mM sodium phosphate pH 8.5 at an enzyme concentration of 10–15 µM. Near-UV and visible CD spectra were recorded on a Jasco J-1500 spectropolarimeter, equipped with a Peltier-type temperature controller, as previously described [21,22]. In brief, 1 mg/mL TgCBS variants’ near UV-Vis (250–600 nm) spectra were collected in a 1 cm path length quartz cuvette at a scan speed of 50 nm/min in 20 mM sodium phosphate pH 8.5 at 25 °C. A minimum of three accumulations were made for each scan, averaged, and corrected for the blank solution of the corresponding buffer.

Thermal unfolding was monitored by recording ellipticity the signal at 222 nm in a temperature range between 20–90 °C (scan rate 90 °C/h). Protein concentrations were 0.2 mg/mL and measurements were performed using quartz cuvettes with a path length of 0.1 cm.

### 4.4. Gel Filtration

The oligomeric state of TgCBS variants was investigated via gel filtration using a GE Healthcare Superdex 200 10/300 GL column in 20 mM sodium phosphate buffer pH 8.5, 150 mM NaCl, and 0.1 mM DTT. The calibration curve was obtained using the high molecular weight gel filtration calibration kit (GE Healthcare), following protocols in [36,37,38].

### 4.5. Trypsin Cleavage of the Enzyme

Purified TgCBS and TgCBS R353* (100 μg) were cleaved with trypsin at 1:200 (*w*/*w*) ratio at 25 °C, in 20 mM sodium phosphate buffer pH 8.0 [39]. The proteolytic cleavage was stopped in 15 μL aliquots at time intervals of 0, 1, 5, 10, 20, 40, 60, 100, and 120 min by boiling the sample for 5 min. The aliquots were then subjected to SDS–PAGE and Western blot analysis by using the monoclonal anti-polyhistidine peroxidase conjugate (Sigma-Aldrich, Milan, Italy dilution 1:2000) against the N-terminal His-tag. The time-course of enzyme activity during trypsinolysis was determined using the CBL-LDH assay as described previously. To evaluate the sizes of the proteolytic fragment, 250 μg of TgCBS were incubated with trypsin for 120 min. The reaction was stopped by adding a two-fold weight excess of soybean trypsin inhibitor (Sigma) and the proteolysis product was loaded on a Superdex 200 10/300 GL column.

### 4.6. Ferguson Plot

The oligomeric state of native TgCBS R353* was determined by Ferguson plots [27,36,40]. Briefly, aliquots of purified TgCBS R353* (10 μg/lane) were electrophoresed through non-denaturing 8, 9, 10, and 12% polyacrylamide gels, and protein mobility (Rf) was calculated for each sample relative to the tracking dye. The retardation coefficient (Kr) was determined from the slope of the plot of 100 [log (Rf × 100)] against the acrylamide concentration. The Ferguson plot was constructed by plotting the log of the negative slope against the log of molecular mass to obtain a standard curve [27]. α-Lactalbumin (14.2 kDa), carbonic anhydrase (29 kDa), chicken egg albumin (45 kDa), and bovine serum albumin (monomer 66 kDa and dimer 132 kDa) were used as molecular mass standards.

### 4.7. Differential Scanning Calorimetry

The DSC (differential scanning calorimetry) experiments were carried out in a nano-DSC calorimeter (TA instruments, New Castle, DE, USA) with a cell volume of 300 μL. Experiments were performed in a 15–120 °C range at a 0.5–1.5 °C/min scan rate. Protein samples contained 30–80 μM TgCBS in 20 mM sodium phosphate pH 8.5. The reversibility of thermal transitions was checked by performing reheating runs after the transitions were completed. Analysis of DSC transitions was performed in all cases using a two-state irreversible denaturation model as described in [4,31]. Data are presented as the mean ± standard error of the mean (SEM) of three repetitions using at least three independently purified protein batches. Theoretical ΔH values were evaluated using the correlation between unfolding enthalpies (at 60 °C) and heat capacities on the protein size (in a number of residues) as described elsewhere [4,32].

### 4.8. Structural Analysis of TgCBS and Its Homologs

The structural analysis and graphical representation of TgCBS and its functional homologs were performed with Pymol [41] and UCSF Chimera [42]. The identification of the main protein cavities was done with Computed Atlas of Surface Topography of Proteins (CASTP v3.0) [43]. The default radius probe used in these calculations was 1.4 Å. The atom coordinates of the target proteins were obtained from the PDB Database (http://www.rcsb.org) (PDB IDs: 6XWL (TgCBS), 4L0D (HsCBS), 3PC2 (DmCBS), and 5OHX (AmCBS).

## Figures and Tables

**Table 1:** Steady-state kinetic parameters of TgCBS full-length and TgCBS R353* for canonical reactions ^a^.

Kinetic Parameter	Full-Length ^b^	R353*
**L-Ser + L-Hcys** **→** **L-Cth + H_2_O**		
*k*_cat_ (s^−1^)	6.3 ± 0.4	5.7 ± 0.8
*K*_m_^L-Ser^ (mM)	0.42 ± 0.04	0.6 ± 0.1
*K*_m_^L-Hcys^(mM)	0.23 ± 0.03	0.20 ± 0.04
*k*_cat_/*K*_m_^L-Ser^ (mM^−1^ s^−1^)	15 ± 2	10 ± 2
*k*_cat_/*K*_m_^L-Hcys^ (mM^−1^ s^−1^)	27 ± 4	29 ± 6
*K*_i_^L-Hcys^ (mM)	1.0 ± 0.1	1.3 ± 0.4
**L-OAS + L-Hcys** **→** **L-Cth + acetate**		
*k*_cat_ (s^−1^)	5.5 ± 0.1	5.6 ± 0.6
*K*_m_^L-OAS^ (mM)	1.3 ± 0.2	2.8 ± 0.5
*K*_m_^L-Hcys^ (mM)	0.20 ± 0.05	0.27 ± 0.04
*k*_cat_/*K*_m_^L-OAS^ (mM^−1^ s^−1^)	4.2 ± 0.7	2.0 ± 0.4
*k*_cat_/*K*_m_^L-Hcys^ (mM^−1^ s^−1^)	28 ± 7	21 ± 4
*K*_i_^L-Hcys^ (mM)	1.4 ± 0.2	1.4 ± 0.2

^a^ Reactions were carried out in 50 mM MOPS, 50 mM bicine, 50 mM proline buffer pH 9 (pH optimum) containing 0.2 mM NADH, 2 μM LDH, 1.5 μM CBL, and 0.1–30 mM L-Ser (or 1–100 mM L-OAS), 0.05–10 mM L-Hcys, and 0.2–2 μM TgCBS full-length or R353* at 37 °C. The data were fit as described in the Materials and Methods section. Reported values represent the means ± S.E.M of three to five repetitions using at least three independently purified protein batches. ^b^ From reference [21].

## Data Availability

Data are available upon request from the corresponding author.

## Supplementary Materials

The supporting information can be downloaded at: https://www.mdpi.com/article/10.3390/ijms23158169/s1. References [4,22,31,44] are cited in the supplementary materials.
