# Supplementary material for: Insights into Domain Organization and Regulatory Mechanism of Cystathionine Beta-Synthase from Toxoplasma gondii
Source: Int J Mol Sci. 2022 Jul 25;23(15):8169. doi: 10.3390/ijms23158169 (PMC9331509; doi:10.3390/ijms23158169)
Supplement: Supplementary file 1 [file ijms-23-08169-s001.zip › ijms-1832234-supplementary.pdf]

Supplementary Material

Insights into Domain Organization and Regulatory Mechanism of Cystathionine Beta-Synthase from *Toxoplasma gondii*

Carolina Conter [1], Silvia Fruncillo [1,†], Filippo Favretto [1], Carmen Fernández-Rodríguez [2], Paola Dominici [1], Luis Alfonso Martínez-Cruz [2] and Alessandra Astegno [1,*]

[1] Laboratory of Biochemistry, Department of Biotechnology, University of Verona, Strada le Grazie 15, 37134 Verona, Italy
[2] Center for Cooperative Research in Biosciences (CIC bioGUNE), Basque Research and Technology Alliance (BRTA), Bizkaia Technology Park, Building 801A, 48160 Derio, Spain
* Correspondence: alessandra.astegno@univr.it
† Current address: Manchester Institute of Biotechnology, University of Manchester, 131 Princess Street, Manchester M1 7DN, UK.

List of the material included:

Table S1. Thermal denaturation parameters for CBS from different organisms as determined by differential scanning calorimetry (DSC).

Figure S1. Properties of TgCBS R353*.

Figure S2. Main cavities present in CBS enzymes containing a Bateman module.

Table S1. Thermal denaturation parameters for CBS from different organisms as determined by differential scanning calorimetry (DSC).

CBS	T_m (°C)	ΔH (kcal/mol)	Ref.
TgCBS wild type	51.1 ± 0.2	80 ± 9	Present work
	42.3 ± 0.7	16 ± 5	Present work
TgCBS R353*	52.7 ± 0.1	67 ± 2	Present work
	39.2 ± 0.1	21 ± 7	Present work
HsCBS	52.8 ± 0.1	80 ± 5	[2,3,4]
	71.4 ± 0.1	223 ± 19	
HsCBSΔ414-551	~ 56	< 9.5	[2,3,4]
	71.4 ± 0.1	223 ± 19	
DmCBS	70.8	388 ± 3	[2]

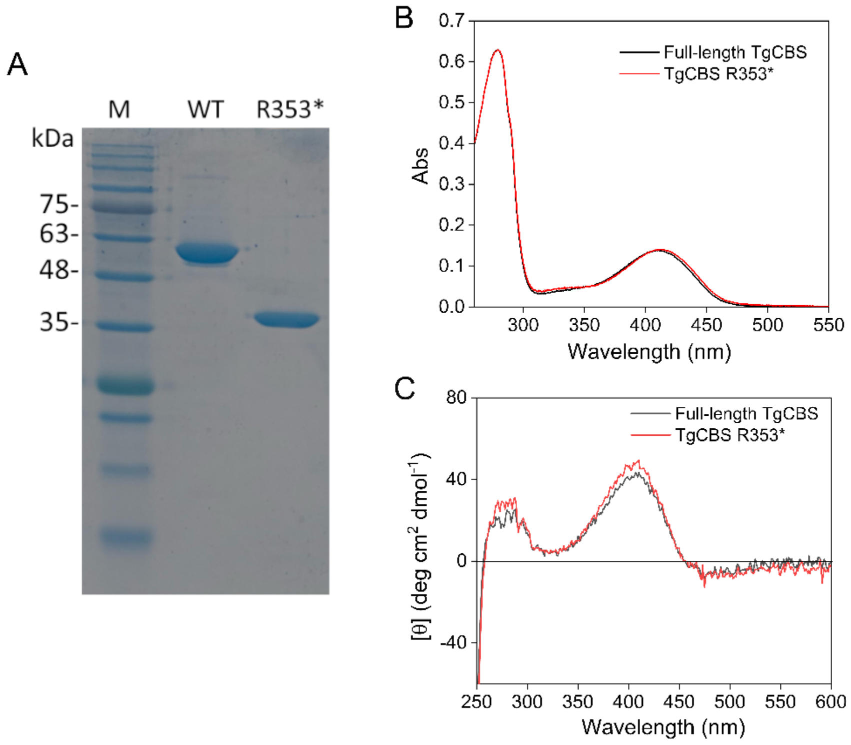

Supplementary Figure S1. Properties of TgCBS R353*. (**A**) 12% SDS–PAGE analysis of purified recombinant TgCBS variants. Lane M, protein marker. (**B**) UV-visible absorption spectra of 15 μM purified full-length TgCBS (black) and TgCBS R353* (red) recorded in 20 mM sodium phosphate buffer pH 8.5. (**C**) CD spectra in the near-UV-visible region of 1 mg/mL full-length TgCBS (black) and TgCBS R353* (red) in 20 mM sodium phosphate buffer pH 8.5.

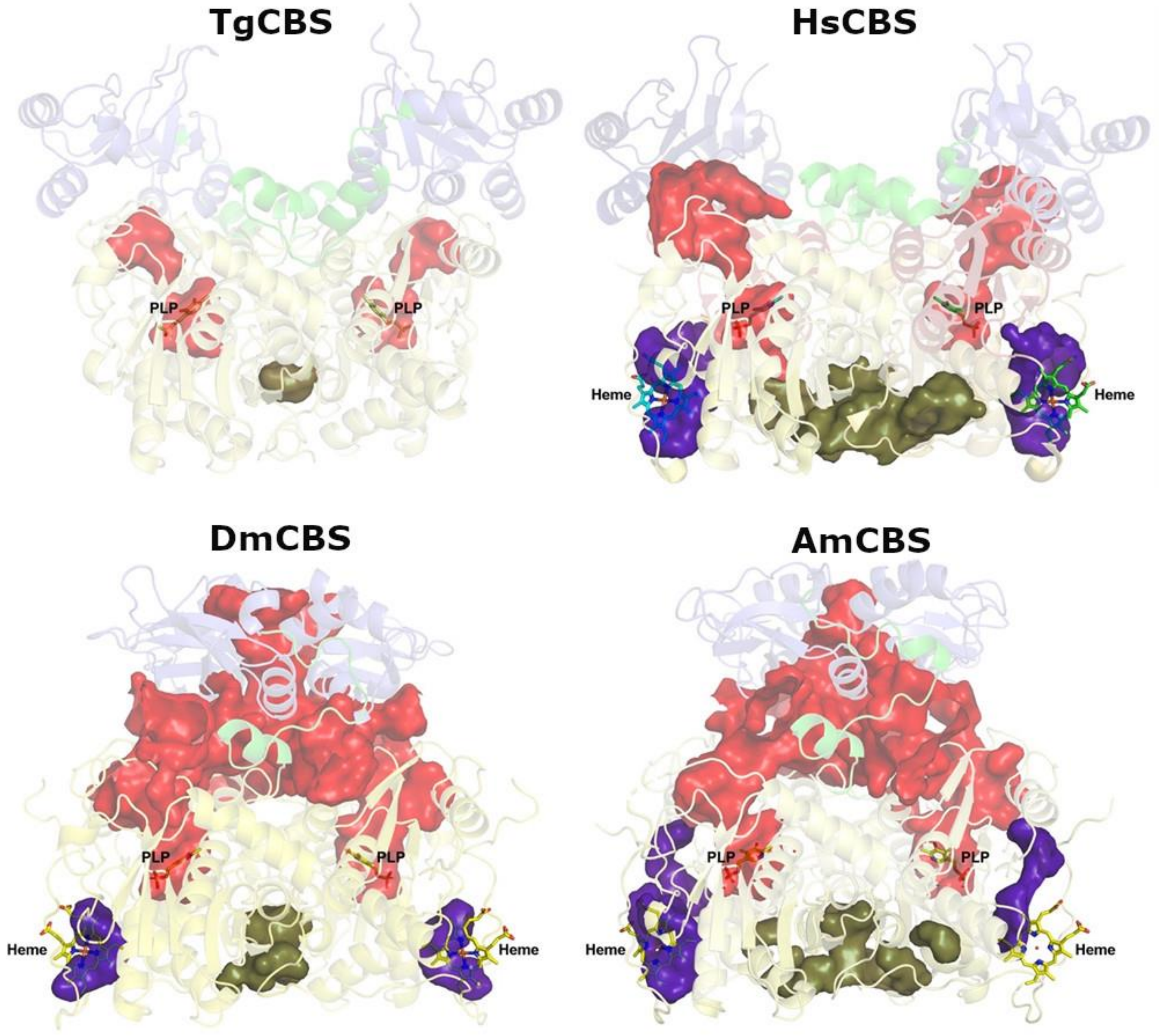

Supplementary Figure S2. Main cavities present in CBS enzymes containing a Bateman module. The figure depicts the three-dimensional structure of the dimeric species of all known CBS enzymes containing a Bateman module, for which the crystal structure is available in the protein databank: *Toxoplasma gondii* CBS (TgCBS, PDB ID 6Z3S) (top, left); *Homo sapiens* CBS (HsCBS, PDB ID 4L0D) (top, right); *Drosophila melanogaster* CBS (DmCBS, PDB ID 3PC2) (bottom, left) and *Apis mellifera* (AmCBS, PDB ID 5OHX) (bottom, right). PLP and Heme molecules are in sticks. The secondary elements configuring the catalytic core, the interdomain linker, and the Bateman module in each protein monomer are colored in yellow, green and dark blue ribbons, respectively. The cavities are represented as colored surfaces. Of note, the catalytic cavity (in red) in TgCBS is less voluminous than in HsCBS (in red, upper, right panel) (see also Figure 7). Remarkably, the equivalent cavities in DmCBS, (bottom, left panel) and in AmCBS, (bottom, right panel), are significantly wider and extend towards the corresponding Bateman modules (in blue ribbons), thus configuring potential alternative entry for substrates. The wider access to the catalytic cavity found in insects CBS, explains

the higher activity of these enzymes with respect to the human species. As shown, the Bateman modules (blue ribbons) are separated from each other and locate above the entrance of the catalytic cavity in TgCBS and HsCBS. This type of arrangement is designed as the basket-like conformation. In contrast, the flies and honeybee dimers, show interacting Bateman modules (blue ribbons), that associate in a disk-like assembly known as "CBS module". We recently baptized this type of dimeric arrangement as "bollard-like" CBS dimer and corresponds to a high activity CBS conformation. In humans, the bollard-like CBS dimer is only adopted upon binding of AdoMet at the Bateman module, whereas in insects, this CBS assembly represents the sole conformation of the enzyme (explaining why flies and honeybee CBSs are constitutively activated). Surprisingly, TgCBS is also very active despite adopting the basket-like arrangement found in the basal (poorly active) conformer of HsCBS. We recently explain such high activity on the basis of a displacement of the complementary Bateman modules toward the central space existing between the complementary subunits [1]. The surface of the intersubunit cavities found in each CBS enzyme is colored in green, whereas the heme-binding cavity (absent in TgCBS) is colored in violet.

References

1. Fernández-Rodríguez, C.; Oyenarte, I.; Conter, C.; González-Recio, I.; Núñez-Franco, R.; Gil-Pitarch, C.; Quintana, I.; Jiménez-Osés, G.; Dominici, P.; Martinez-Chantar, M.L.; et al. Structural Insight into The Unique Conformation of Cystathionine Β-Synthase from *Toxoplasma Gondii*. *Comput. Struct. Biotechnol. J.* **2021**, *19*, 3542–3555. https://doi.org//10.1016/J.Csbj.2021.05.052.
2. Majtan, T.; Pey, A.L.; Fernández, R.; Fernández, J.A.; Martínez-Cruz, L.A.; Kraus, J.P. Domain Organization, Catalysis and Regulation of Eukaryotic Cystathionine Beta-Synthases. *PloS ONE* **2014**, *9*, E105290. https://doi.org//10.1371/Journal.Pone.0105290.
3. Pey, A.L.; Majtan, T.; Sanchez-Ruiz, J.M.; Kraus, J.P. Human Cystathionine Β-Synthase (Cbs) Contains Two Classes of Binding Sites For S-Adenosylmethionine (Sam): Complex Regulation of Cbs Activity and Stability by Sam. *Biochem. J.* **2013**, *449*, 109–121. https://doi.org//10.1042/Bj20120731.
4. Pey, A.L.; Martínez-Cruz, L.A.; Kraus, J.P.; Majtan, T. Oligomeric status of human cystathionine beta-synthase modulates AdoMet binding. *FEBS Lett.* **2016**, *590*, 4461–4471. https://doi.org/10.1002/1873-3468.12488.
